# The Holloway Sanitorium for the Insane at Virginia Water, Egham

**Published:** 1889-02-09

**Authors:** 


### The Holloway Sanatorium for the Insane at Virginia Water, Egham.

This asylum owes its existence to the munificence of the late Mr. Thomas Holloway. It was this gentleman's intention to build and fully equip the institution as a hospital for the treatment of insanity. It was to have been fairly launched on its career of usefulness, and then, we believe, Mr. Holloway's idea was that the asylum was to be self-supporting—meaning, presumably, that although some patients would be admitted at payments less than the actual cost, others, in better circumstances, would pay a little more, and so equalise matters. At the same time, the accommodation provided for the latter was understood to be better than they could possibly receive at any private asylum for like sums. Mr. Holloway unfortunately died before the building was finished, and, curiously enough, he left no provision for its completion. His relatives and trustees—Mr. G. Martin-Holloway, Mr. H. Driver-Holloway, and Miss Driver—generously decided to come to the rescue, and finish the institution in the manner originally intended.

The trustees were extremely fortunate in securing the services of Dr. Sutherland Rees Philipps as medical superintendent. That gentleman entered on his duties in June, 1884, bringing long and varied experience to bear on the most arduous task ever entailed in organising and opening a large establishment for the insane. It did not escape Dr. Philipps's observation that the building was badly planned, having at least one serious blemish and a good many minor ones. The absence of corridors of communication made the portion of the building intended for the accommodation of patients almost useless. Additional staircases were needed; airing courts had to be enclosed; drying-grounds for the laundry walled in; open spaces had to be covered in; heavy partition walls had to be pulled down to admit light and air; mess-rooms for the staff had to be added; and even the medical superintendent's residence was converted out of a part of the building intended for a totally different purpose. We are not called upon in this article to criticise the building; if so, we should feel inclined to look at it from three different points: (1) What it was originally; (2) what it is at present—that is, since many of Dr. Philipps's ideas have been carried out; and (3) what it will be when he has finished his scheme of reconstruction. We may return to this on some future occasion, and in the meantime we merely say that the medical superintendent has surmounted difficulties that would have damped the energies of most other men. In addition to the above alterations, gas-works have been built for providing gas for the wards and for the officers' rooms; while the electric light is used in the dining-hall, the recreation-room, and entrance. Lastly, the sewage and drainage works have been thoroughly overhauled, and are now well abreast of all the demands of sanitary science.

The institution was registered as a hospital on June 13th, 1885, and two days afterwards it was formally opened by the Prince and Princess of Wales. The reception of patients was begun immediately after the opening, and at the close of its first complete year of existence it contained 110 patients. During 1887 there were 95 admissions, and the year closed with 149 patients on the register. At the date of writing this article the sanitorium is almost full, there being only ten vacancies for ladies and twenty for gentlemen; but a detached building for some twenty or thirty cases is ready for occupation.

It is extremely gratifying to notice that paralytics and such cases as are often refused at other hospitals are here admitted, and it is to be earnestly hoped that nothing will ever prevent these cases being taken in and treated. Of course the percentage of cures will be reduced, but the usefulness of the hospital will be increased, and that is the chief thing to look at. We note also that cases not under certificates are admitted—that is, they are really boarders in the institution, and may leave when they like. We doubt whether accommodation for this class of patients is much wanted in England.

Some patients are received free of charge, others pay as low as 2s. 6d. a-week, and, apparently, quite a number are kept at 25s. a week. About two guineas a-week would seem to be an ordinary charge.

The amusement and recreation of the patients are provided for in the most liberal manner. In one year upwards of £500 were spent in this way.

In private asylums, and in public ones too, there is always some difficulty in finding employment at once suitable and safe for the inmates. At Virginia Water various occupations are fostered. They embrace gardening and in-door work, and we are told that "further development will include special arrangements for increasing and diversifying the useful occupation and the amusement of the patients."

On turning to the expenditure column of one of the reports we find that the drug bill amounted to £65, so it is clear the medicinal treatment of the patients is by no means overlooked by Dr. Philipps and his colleagues.

An innovation—at least, so far as our knowledge of English asylums goes it is an innovation, although common in America—consists in allowing patients' friends to reside with them. In some cases we hear that visitors have remained weeks, and even months, with their afflicted friends. Another point worthy of comment is that Dr. Kirkbride's idea of employing lady and gentlemen companions has been adopted here, and the practice is said to answer admirably. In one of his reports Dr. Philipps mentions that it was the intention of the board of management to appoint a permanent chaplain, who would reside in the neighbourhood, and visit the asylum daily. "If the right man is chosen, the appointment will be a very valuable one for the hospital. To satisfactorily fill such a post, the chaplain must be a man of broad and liberal views, of some musical ability, genial in manner, and earnest in his work; one who can gain the confidence and respect of patients and staff." So writes Dr. Philipps. We agree with him, and hope he has been successful in finding such a heavenborn colleague as he looked for. If so, and if he would publish his method of obtaining such a man, he would be conferring a real favour on many of his brother superintendents.

The surroundings of a patient may be presumed to have an important effect on him, tending to the recovery of curable cases, and to the contentment of such as may be incurable. This matter has been most carefully attended to at Virginia Water. A visitor is at once struck with the refined taste shown in the furnishing and decoration of the wards; in fact, they do not look like wards, as ordinarily understood. That part of the institution occupied by patients resembles a succession of drawing-rooms more than anything else. Without exception the rooms are comfortable, and many of them are luxurious. The recreation-hall is, if anything, rather over-decorated.

We were much gratified by our personal inspection of this hospital, and it is our opinion that it has at once sprung to the front rank among kindred institutions.

